# Cholera Infantum—Read before the Erie County Medical Society, January, 1862

**Published:** 1862-03

**Authors:** Henry Nichell


BUFFALO

### Medical and Surgical Journal

AND REPORTER.

VOL. I. MARCH, 1862. NO. 8.

### ORIGINAL COMMUNICATIONS.

ART. I—*Cholera Infantum—Read before the Erie County Medical Society, January*, 1862, *by* HENRY NICHELL, M. D. *Published by vote of the Society.*

[CONCLUDED.]

SYMPTOMS.—In acute cases of a mild character, the attack is, as a rule, preceded for a series of hours or days, by diarrhœa; after such time, moderate vomiting is apt to occur; the latter symptom, however, may, in the course of a day, or two, become less frequent, and finally cease, leaving only the alvine dejections behind. Sometimes there may be but a slight diarrhœa at the commencement, with occasional vomiting and some febrile action, till, after a while, both excessive vomiting and copious watery purging suddenly appear, or, the attack may, from the beginning, occur with great vehemence, without any precursory symptoms. In such cases, vomiting continues almost incessantly with profuse watery diarrhœa and paroxysms of pain in the abdomen, the intervals of which are marked by great languor and distress. This condition is soon followed by alarming exhaustion and fatal collapse; the pulse becomes wiry and almost imperceptible, the face and extremeties cold, the countenance deathly pale and contracted, the eyes sunken; the little sufferer lies almost motionless, while, in other cases of this character, violent convulsions take place, until, at last, death approaches, in most instances, at a period varying from five or six hours to a few days. There are cases in which far less vomiting occurs, but very frequent distressing attempts "at vomiting," together with a certain convulsive motion of the abdominal walls, which is found on placing the hand slightly over this region when the stomach is acting in the manner described. Cases presenting this character are generally severe; it is often indicative of disease of the brain. At the beginning, the discharges consist of the ordinary fœcal matter, although more freely mixed with watery fluid than common; these are followed sooner or later by frothy serum, colored with bile, or of a nearly colorless fluid containing minute shreds or flocculi of intestinal mucus. In most cases, at the same period, there are evidences of acidity in the evacuations, while in the further course of the disease, they are apt to present a great variety of more or less important changes in their character, thus they may become serous, mucous, bile-colored, chalklike, muco-sanguineous, and purulent, alternately. They are, throughout the disease, either inodorous, or acid, sometimes highly offensive and putrid in smell. The pulse mostly is quick, small, and in certain cases, somewhat tense—occasionally, it is found also sharp and frequent; in cases running a more or less protracted course, it is weak and frequent, and sometimes corded. The head and abdominal region are generally more or less hot during the early stages. In the progress of the complaint, there is a well-marked decrease of the general temperature of the body, except the abdomen, which, on the contrary, seems to be immensely increased in temperature. Urine is scanty, and there is generally great thirst.

An eruption upon the chest, of an immensity of watery vesicles of a very minute size, as has been observed by Drs. Dewees, Condie, Rush, and others, deserves to be mentioned, since it probably appears in the later stages of the complaint. Although Dr. Dewees considered it an invariably fatal symptom, there is at least no less celebrated authority than Dr. Condie, who contradicts it by fact. He says: "That he has, in many instances, known the patient to recover, even when the eruption has been the most extensive and distinct."

In confirmed cholera infantum, the brain often appears to be affected, either its substance or its meninges. There may exist congestion, or even inflammatory action, or any of their consequences. In acute cases, delirium may occur early in the disease. The child frequently appears quite restless, his eyes are wild and injected, there is aversion from light along with other head symptoms. Several years since, I observed a little girl two years of age, who, suffering from this complication, was placed on her parents, large bed, rolling herself constantly with much power in every direction, till cold applications were made to the scalp, which quieted her almost immediately. Cholera infantum shows a decided tendency to assume a more or less protracted character, when emaciation goes on very rapidly; the muscles of the little sufferer become flabby, the skin appears dry and cool and of a peculiar dirty or even brownish hue; the face shrivelled and pale, presents, in such instances, often just the miniature appearance of old age. The eyes are hollow and glassy, the mouth most frequently becomes aphthous; the abdomen is either tympanitic or sunken and the discharges are dark colored or chalk-like and very offensive. The utmost exhaustion prevails, together with partial stupor and signs of paralysis, until at last the little patient either dies comatose, or during a fit of convulsions. In addition to the affections of the brain and liver, which may be developed in the course of cholera infantum, various other accidental disease are occasionally observed to coexist, as acute and chronic bronchitis, pneumonia, whooping-cough, stomatitis, measles, scarlatina, etc., some of which, may likewise appear as sequels of the complaint.

*Treatment.*—I shall call your attention to the treatment of the disease, the causes, anatomical characters, pathology and symptoms of which, I have already pointed out, in some measure. Although I am unable to offer any new means for the relief or cure of the complaint in question, yet I believe that a further establishment of the efficacy of already known medicinal agents will appear at least of some practical value to the profession. In the treatment of cholera infantum the efforts of the practitioner should principally be directed to remove the child from those active morbific causes by which the disease has been produced, and may be kept up for an indefinite period. Without fulfilling this chief indication so far as experience has proved, no permanent cure can be effected. Even if the patient has apparently beencured, the disease is apt to return several times, in spite of the most rational treatment, and may strongly manifest the unpleasant and changeable characters of temporary relief, with alternations of perilous relapse Should circumstances render it practicable to carry the suffering child into the fresh and pure air of the country a happy change in the disease would likely result. In cases however were the removal to a more airy and healthy country place cannot be effected, the patient should at least be exposed frequently, or if it is possible, daily, to the salutary influences of in pure air, the most healthy parts of the city, and kept there for a reasonable time. When at home the utmost care should be taken to keep the apartments perfectly dry, clean, and well ventilated. The clothing should always be in accordance with the temperature of the air, and neither too warm, nor too heavy, so as to oppress the infant. The patient should by no means be permitted to sleep on a feather bed, as it very naturally serves to accumulate the heat, which of course will greatly add to the severity of the disease; on the contrary he should rest on a matrass, with but a light and loose covering. The daily immersion in a warm or tepid bath, followed by frictions over the entire cutaneous surface, with a soft and dry cloth, as recommended by various writers, is very useful as long as prostration has not already commenced. Infantile diet, is, as has been remarked, a most important subject in cholera infantum, and it is the stern duty of the attending physician to urge the strictest compliance with dietetic laws and principles, as errors in diet during the hot season in this climate, are among the most frequent exciting causes of the disease. The food of the infant should be the mother's milk, as long as the latter in no wise appears to be deteriorated. A certain regularity in nursing the little patient is an important item in the treatment. Should, however, for any reason a substitute be required, fresh cow-milk, diluted with water, will answer the purpose. According to Simon and West, woman's milk contains 33 parts of caseine, 24 of butter, and 46 of sugar, in 1000; cow's milk 42 of caseine, 28 of butter, and 24 of sugar, in 1000. Cow's milk should, therefore, be largely diluted with water to reduce the caseine and butter in imitation of human milk. Also the use of gelatine added to pure or diluted milk, as recommended by the experienced Dr. Steward, may be substituted for the food which nature supplies. Occasionally neither the milk from a mother's breast or cow, will be retained in the stomach, being almost instantly ejected; in such instances abstaining from its use as food, for at least from twelve to twenty-four hours, and substituting toast-water and mutton tea, will generally prove beneficial. At the commencement of the complaint, especially in cases where vomiting is severe, cold toast-water only should be permitted as a drink, given in small quantities, every ten minutes, which, according to my experience, will in the vast majority of cases be retained. Should any degree of exhaustion or prostration become evident, the administration of diffusible stimulants will be indicated. The addition of one-half tea-spoon brandy to toast-water, given every hour or oftener, as circumstances may require, will frequently be of service. Experience has shown that the use of chicken or mutton tea, as nutriment, is but rarely ejected from the stomach, or found to increase the alvine evacuations. Occasionally if the patient should appear eager for some animal food, as for instance a piece of chicken-flesh, or dried salted beef or ham, he should be satisfied in this respect. The use of vegetable articles, however, either of a farinaceous or starchy nature, I would not recommend on account of its well known tendency of turning acid, which but too often has led to bad consequences. The chief indications in the treatment of cholera infantum are—to control vomiting and purging—to create a free biliary secretion—to support the powers of life, so as to prevent either a rapid collapse in the acute form, or any considerable degree of exhaustion and prostration in the more chronic cases, and finally—to remove the causes of the disease so far as possible. In the commencement of the attack the irritability of the stomach should be allayed without any loss of time. If the disease presents a mild character, and there exists but moderate vomiting, together with more or less acidity of the primæ viæ, the employment of some alkali appears to be rational. Lime water and fresh cow-milk, half a tea-spoon full of each, mixed together, and given every half hour, or oftener, for several hours, will usually suffice to arrest it. In addition to this, in order to correct the frequency and character of alvine dejections, I have been in the habit for many years of prescribing almost invariably the following:

℞ Calomel gr. ii.
Pulv. Opii.
Pulv. Ipecac, aa gr. i.
Magnes. carbon, gr. xii.
M. f. pulvis.
Divide into twelve parts for a child of two years.

One powder every two hours, to be continued as long as the alvine discharges are profuse and watery. When, however, they become less frequent and assume a more healthy character, the powder should be administered every three, four, six or eight hours, or even suspended.—The attending physician should obtain full information of the effect of remedies, containing opium at least two or three times daily, in order to be able to forbid its further use, should it for any reason be counter-indicated. Many authorities object to the employment of opium in the diseases of early infancy. Indeed the use of this powerful remedial agent in young infants requires the utmost caution on account of its peculiar effects on the brain, which may most unexpectedly result in profound sopor, convulsions and sudden death. Dr. Steward says: "At the commencement of the disease it should never be employed," while on the other hand Prof. Jacobi uses the following language: "I scarcely ever treat either an acute or chronic catarrh of the intestinal canal without opium, as the occurrence of the affection without greatly increased motion, is not possible. At the same time the remedy has a decided effect on the secretion of the follicles of the mucous membranes and glands." In the report of the Nursery and Child's Hospital, the following is said in regard to the treatment of cholera infantum: "The old idea of congestion or torpidity of the liver, so as to indicate mercurial treatment has been shown to be erroneous by the post-mortem examinations made in this institution in cases of cholera infantum. The mixture employed to check the disease contained for the most part some form of opium. The opiate treatment proved very effectual in quieting the bowels, but it was used cautiously, and not at all in the advanced stages of the disease, when cerebral symptoms were threatening." From this, the addition of calomel to opiates does not appear absolutely necessary, but on the other hand, the mercurial is recommended by the most satisfactory results. When the vomiting is violent and frequent, creosote is a very reliable remedy. The addition of two drops of creosote to a fluid ounce of lime-water, given with milk, in half tea-spoon quantities of each, every one or two hours, or even after each vomiting, will prove beneficial in many cases. At the same time a large sinapism should be applied over the abdomen to produce some counter-irritation. Should creosote, however, fail to control this symptom, I have mostly succeeded with the solution of the acetate of lead, as recommended by Dr. Condie, or by the employment of sub-carbonate of bismuth, in from three to five grain doses, given every two hours, for a day or two, by which also the discharges from the bowels became altered and less frequent. Should vomiting still persist, while great exhaustion and rapid collapse are threatening, very small pieces of ice should be placed on the patient's tongue every eight or ten minutes, and brandy and toast-water mixed together should be given in tea-spoon quantities, from time to time. Stimulating injections containing a tea-spoon full of brandy may be made use of under these circumstances. As there is no doubt the patient is suffering from pain in the bowels at times, either at the commencement or during the further course of the complaint, the application of warm poultices over the abdomen for a day or two appears rather preferable to the employment of leeches, which but too often will induce exhaustion and even collapse. To arrest profuse watery discharges from the bowels acetate of lead seems to be indicated: it is a most important remedy in cholera infantum, especially in those cases where the watery dejections are very copious and frequent, and the patient is already debilitated; there is no other remedial agent within the entire materia medica which will arrest these discharges so promptly; it should be suspended as soon as the discharges lose their watery character. After this, chalk-mixture with the addition of tannin and tincture opii, or the gallic acid, may be employed for some time to prevent any relapse of the disease. In the more advanced stages when the continuance of the discharges is gradually followed by a state of prostration and more or less emaciation, astringents in combination with tonics, as tannin quinine, etc.should be used. In chronic cases where the discharges are found entirely inodorous, consisting of a mixture of jelly-like mucus and watery fluid, at times perhaps stained with blood, together with absence of pain in the abdomen, the spirits of turpentine will frequently prove a very valuable remedy when perseveringly employed. In very obstinate instances of diarrhœa, characterized by absence of pain in the bowels, after many other means have been tried in vain, the liquor persesquinitrate of iron may be given in five drop doses, three times daily, and gradually increased to fifteen, to a child of two years.

I will not trespass upon your time in treating the complications of the disease.

*Corrections.*—Page 199, of the February number, commencing at seventh line from the top read: "Inasmuch as it appears that the process of teething has little if anything to do with it. Since it is a fact that cholera infantum while making its attacks only during a certain age of infancy, often occurs however previous to the period when the actual protrusion of teeth usually takes place.—Secondly,—The period of teething includes also the time during which another physiological process occurs, of much greater influence in the production of the disease, viz., the developement of the muciep arous follicles."

---

*Costello's Cyclopædia of Surgery.*—We read in the *Gazette Hebdomadaire* that this work, lately completed, was destroyed by the fire which broke out at the printing office of Messrs. Taylor and Greening, in London. Luckily 500 copies had lately been removed.

IN THE *Dublin Medical Press,* Dr. Thomas Davis describes "a case of extra-uterine fœtation of eight years and three months standing, counting from the expected period of delivery. The fœtus was successfully removed by operation.—*British Med. Jour.*

